# Sustained low-efficiency diafiltration is superior to hemodialysis in promoting renal function recovery in elderly wasp sting victims with stage III acute kidney injury: a retrospective study

**DOI:** 10.1080/0886022X.2019.1655449

**Published:** 2019-09-05

**Authors:** Yan-Yan Deng, Jian-Ming Shen, Ya-Ni Mao, Rong Gou, Wen-Wen Li, Ting-Ting Ye

**Affiliations:** Department of Nephrology, Renmin Hospital, Hubei University of Medicine, Shiyan, China

**Keywords:** Sustained low-efficiency diafiltration, hemodialysis, acute kidney injury, wasp stings, renal function

## Abstract

**Objective:** To study the efficacy and safety of sustained low-efficiency diafiltration (SLEDf) *versus* hemodialysis (HD) for patients with wasp stings who developed stage III acute kidney injury (AKI).

**Methods:** We retrospectively analyzed the clinical data of consecutive patients who developed AKI following wasp stings. All eligible patients received renal replacement therapy in combination with hemoperfusion. Thereafter, blood purification therapy and HD were performed with a volumetrically controlled machine and 1.7 m^2^ surface, Fresenius Polysulfone HD filter and SLEDf was undertaken with a volumetrically controlled machine and 1.3 m^2^ surface, Fresenius Polysulfone HD filter.

**Results:** Forty patients developed stage III AKI following wasp stings, including 14 patients that received SLEDf and 26 patients underwent HD. Thirteen patients were aged less than 60 years and underwent HD (group I), 27 patients were aged at least 60 years, including 13 patients undergoing HD (group II) and 14 patients receiving SLEDf (group III). Groups I and II completed 150 and 162 sessions of HD, respectively, and group III completed 156 sessions of sustained low-efficiency blood purification therapy, including 50 sessions of SLEDf. The time to return to normal serum creatinine levels was 38.8 ± 2.7 days for group I, 47.2 ± 5.3 days for group II, and 39.2 ± 3.3 days for group III. A statistically significant difference was observed in time to normal serum creatinine levels among the three groups.

**Conclusion:** Elderly wasp victims have more severe illness than younger wasp victims and SLEDf is safe and superior to HD in recovery of renal function of elderly wasp victims.

## Introduction

Wasp stings cause erythema, edema, and local pain in mild cases and generally do not cause any major problems. However, in severe cases, apart from anaphylactic reactions, may lead to acute kidney injury (AKI) or multiple organ failure [1,2]. Epidemiological aspects of wasp stings are not well known. Witharana et al. reported that 2.9% of 322 medical ward admissions were attributable to emergency treatment after wasp or bee stings at a local hospital in Sri Lanka [3]. Prompt renal placement therapy may improve patient prognosis, but currently there is no consensus in the literature on which mode of dialysis is more effective for wasp sting-associated AKI. Dhanapriya et al. [4] reported 11 cases of wasp sting-induced AKI in whom 10 patients received hemodialysis (HD) and 9 patients recovered normal renal function at 24 months of follow-up. A retrospective analysis of 81 AKI patients with multiple wasp stings showed that most patients survived with complete recovery of their kidney function and there was no statistical difference in mortality among the patients receiving different modes of blood purification therapy [5].Cho et al. showed that the prognosis of sepsis patients with AKI undergoing continuous renal replacement therapy correlated with age and survivors were younger than nonsurvivors [6]. However, no literature is available on the prognosis elderly AKI patients *versus* young AKI patients following wasp stings.

Sustained low-efficiency diafiltration (SLEDf) is a hybrid renal replacement technique for hemodynamically unstable adult patients with AKI; it possesses the advantages of conventional HD and slow continuous therapies. Sustained low-efficiency dialysis (SLED) uses the conventional dialyzer to provide sustained renal replacement therapy by reducing the velocity of flow of dialysate and blood. It achieves the therapeutic objective by diffusion, convection to a lesser extent, and ultrafiltration [7]. SLEDf as a novel hybrid renal replacement therapy improves clearance of large molecules and increases clearance rate of solutes on the basis of sustained, slow and low efficiency hemodiafiltration for optimized hemodynamic stability, and minimized solute disequilibrium [8]. The hybrid technique has also been successfully used to treat kidney failure patients due to severe star fruit intoxication [9]. SLED avoids high costs and technical complications and has emerged as a cost effective alternative to continuous renal replacement therapy [2].

In this retrospective single institution study, we reviewed the clinical data of patients with wasp stings who developed stage III AKI and received HD or SLEDf with time to normal serum creatinine as the primary study outcome.

## Patients and methods

### Patients

We retrospectively analyzed the clinical data of consecutive patients who developed AKI following waspstings and sought treatment at our tertiary care Renmin Hospital, Hubei University of Medicine, Shiyan, China between January 2008 and December 2011. AKI was diagnosed and staged according to the 2005 diagnostic and staging criteria by the Acute Kidney Injury Network (AKIN) [10]. Our Department of Nephrology only accepts stage III AKI patients requiring renal replacement therapy. Therefore, the study only included patients who developed stage III AKI following wasp stings.

The study protocol was approved by the local ethics committee at the authors’ affiliated institution (approval No. 2016-052) and patient consent was not required because of the retrospective nature of the study. Patients provided informed consent to HD or SLEDf after the benefits and risks as well as economic cost of each procedure were explained.

### Therapeutic interventions

Conventional therapeutic measures included prompt removal of the stinger, analgesics when necessary, early glucocorticoid therapy and fluid volume correction based on patient’s physical examination.

All patients received blood purification therapy once daily for 2 days along with hemoperfusion for 2 h in each session. The hemoperfusion device (HA330 resin, styrene divinylbenzene copolymers, with a blood flow of 200–250 mL/min, volume of 185 mL; Jafron Biomedical Co., Ltd., Zhuhai, Guangdong, China; http://www.jafron.com/) was used for hemoperfusion [11]. Thereafter, blood purification therapy was performed once every other day. HD was discontinued after patients became polyuric (urine volume > 2500 mL/day or urine volume > 1500 mL/day in patients undergoing blood purification [12]). Hemodialysis was performed with a volumetrically controlled machine (4008B; Fresenius Medical Care, Bad Homburg, Germany) and 1.7 m^2^ surface, Polysulfone hemodialysis filter (F60,Fresenius Medical Care). The bicarbonate dialysate flow rate was 500 mL/min, and the blood flow rate was 180–250 mL/min. Each dialysis session lasted for 4 h.

SLEDf was performed using B. Brown Dialog^+^ on-line Hemodialysis System and 1.3 m^2^ surface, Polysulfone HD filter (F60s, Fresenius Medical Care) at a blood flow rate of 180–200 mL/min. Dialysate flow was set at 200 mL/min. Replacement fluid was prepared using the online configuration, pre-diluted, and set at a flow rate of 100 mL/min. Each dialysis session lasted for 8 h. After four dialysis sessions, patients either continued SLEDf or received HD at the discretion of the attending physician.

### Study outcomes

The primary outcome of this study was time to normal serum creatinine from baseline. Secondary outcomes included intra-hospital mortality rate, cure rate, and the time to polyuria, and Acute Physiology and Chronic Health Evaluation (APACHE)II score at days 3 and 7 post-therapy. Side effects of blood purification therapy were also reported. Clinical cure was defined as absence of renal dysfunction, proteinuria, or hematuria, and improvement was defined as kidney abnormality without HD, including creatinine elevation, proteinuria, or hematuria. Patients without normal serum creatinine (scr) and urine tests at discharge were followed up for 6 months

### Statistical analysis

Data were expressed as mean ± standard deviation ($\bar{x}\pm s$) and analyzed using Stata 12.0 statistical software. ANOVA was used for comparison among three groups and if statistical difference was observed, and Scheff method was used for comparison between two groups. Numerical data were compared using Chi-square test or Fisher exact test. *p* < 0.05 was considered statistically significantly different.

## Results

### Patient demographic and baseline characteristics

Forty patients developed stage III AKI following wasp stings during the review period. Their mean age was 60.5 ± 10.3 years and they included 20 men and 20 women. Patient demographic and baseline characteristics are shown in Table 1. The mean duration from wasp sting to initial blood purification therapy was 96.5 ± 17.3 h (range 69–118 h). Among them, 14 patients received SLEDf and 26 patients underwent HD. Moreover, 13 patients were aged less than 60 years and all of them underwent HD (group I), 27 patients were aged at least 60 years, including 13 patients undergoing HD (group II) and 14 patients receiving SLEDf (group III). Patients in groups II and III were comparable in demographic and baseline characteristics. The arterial pressure and urine volume in groups II and III were significantly lower than group I (*p* < 0.05) while the mean APACHE II score of groups II and III was markedly higher than that of group I (*p* < 0.05). There was no statistical difference in time to initial blood purification therapy among the three groups (*p* > 0.05).

**Table 1.** Patient demographic and baseline characteristics.

Variables	All	HD		SLEDf
		Aged less than 60 years	Aged 60 years or above	
No.	40	13	13	14
Age, years				
Mean (SD)	60.5 (10.3)	49.2 (6.7)	65.5 (3.6)	66.4 (3.5)
Range	34, 73	34, 58	60, 72	60, 73
Male gender, *n* (%)	20	5	7	8
Mean arterial pressure, mmHg, mean (SD)	82.8 (16.4)	92.7 (13.2)	79.4 (12.7)^a^	76.9 (13.1)^a,b^
Urine volume, mL/h	7.2 (3.6)	8.9 (2.7)	6.7 (2.2)^a^	6.2 (2.4)^a,b^
Mean (SD) serum creatine, μmol/L	770.3 (158.2)	676.8 (127.2)	811.1 (136.8)^a^	819.2 (124.1)^a,b^
Blood urea nitrogen, mmol/L	42.0 (6.9)	38.3 (6.2)	43.0 (7.3)^a^	44.5 (7.1)^a,b^
WBC ×10^9^/L	25.6 (3.8)	21.5 (3.3)	26.4 (4.2)^a^	28.6 (5.4)^a,b^
Hemoglobin, g/L	91.3 (16.9)	85.7 (16.1)	92.9 (17.2)^a^	94.9 (18.9)^a,b^
Platelets, ×10^9^/L	81.6 (14.9)	77.3 (11.5)	82.3 (15.2)^a^	84.8 (17.4)^a,b^
Total bilirubin, μmol/L	60.5 (14.3)	55.6 (12.3)	61.6 (13.4)^a^	64.0 (19.5)^a,b^
ALT, U/L	1142 (327)	976 (256)	1146 (310)^a^	1292 (410)^a,b^
LDH, U/L	4724 (1194)	4165 (975)	4888 (1187)^a^	5090 (1342)^a,b^
CK, U/L	17,165 (5878)	13,763 (5903)	17,957 (6036)^a^	19,589 (5512)^a,b^
CK-MB, U/L	701 (198)	628 (169)	722 (201)^a^	750 (247)^a,b^
PT, s	23.1 (2.4)	18.6 (2.1)	24.4 (2.5)^a^	26.1 (3.8)^a,b^
aPTT, s	47.6 (10.7)	42.7 (9.8)	48.3 (11.7)^a^	51.4 (8.8)^a,b^
Mean (SD) APACHE II score	21.6 (2.5)	19.9 (2.0)	22.7 (1.9)^a^	22.2 (2.0)^a,b^
Mean (SD) of organs with failure	4.32 (1.15)	3.69 (0.75)	4.62 (0.96)^a^	4.64 (0.93)^a,b^
Mean (SD) time to initial blood purification, h	96.5 (17.3)	93.7 (14.4)	96.3 (12.5)^a^	99.4 (15.8)^a,b^

aPPT: activated partial thromboplastin time; CK: creatine phosphokinase; CK-MB: creatine phosphokinase-MB; HD: hemodialysis; PT: prothrombin time; SLEDf: sustained low-efficiency diafiltration; WBC: white blood cells.

^a^*p* < 0.05 versus patients receiving HD aged < 60 years; ^b^*p* > 0.05 versus patients receiving HD aged 60 years or above.

### Primary outcome

Serum creatinine at baseline was markedly elevated for all three groups *versus* normal reference values and was significantly higher in both groups II and III than group I (*p* < 0.05) while it was comparable between groups II and III (*p* > 0.05) (Table 1).

**Figure 1.**
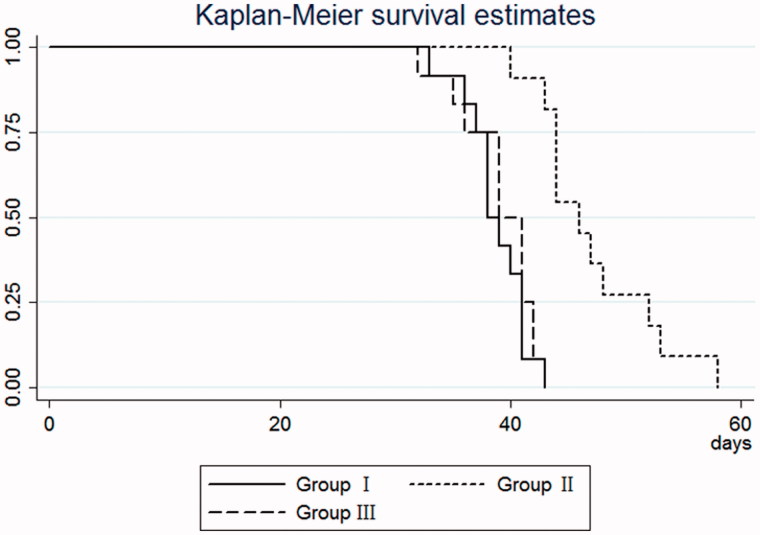
The Kaplan–Meier curve for time to return to normal serum creatinine levels in patients who developed stage III AKI following wasp stings. Group I patients were aged less than 60 years and all of them underwent HD; group II patients were aged at least 60 years, including 13 patients undergoing HD; group III patients were aged at least 60 years and received SLEDf. The time to return to normal serum creatinine levels was significantly longer in group II than group III (*p* = 0.0001) and group I (*p* = 0.0001), respectively. HD, hemodialysis; SLEDf: sustained low-efficiency diafiltration.

Groups I and II completed 150 and 162 sessions of HD, respectively, and group III completed 156 sessions of blood purification therapy, including 50 sessions of SLEDf and 106 sessions of HD. The time to return to normal serum creatinine levels (44–106) μmmol/L) was 38.8 ± 2.7 days for group I, 47.2 ± 5.3 days for group II, and 39.2 ± 3.3 days for group III (Table 2). A statistically significant difference was observed in time to normal serum creatinine levels among the three groups (*F* = 17.20, *p* = 0.005). The time to return to normal serum creatinine levels was significantly longer in group II than group III (*p =* 0.0001) and group I (*p* = 0.0001), respectively (Figure 1).

**Table 2.** Changes in serum creatine and time to normal serum creatinine ($\bar{x}\pm s$).

	Level of serum creatinine (μmol/L)		Time to normal serum creatinine
	Day 3	Day 7	
Group I	452.9 ± 133.1	337.6 ± 70.9	38.8 ± 2.7
Group II	595.8 ± 108.2	457.6 ± 82.9	47.2 ± 5.3
Group III	475.6 ± 84.7.1	370.6 ± 49.0	39.2 ± 3.3

**Figure 2.**
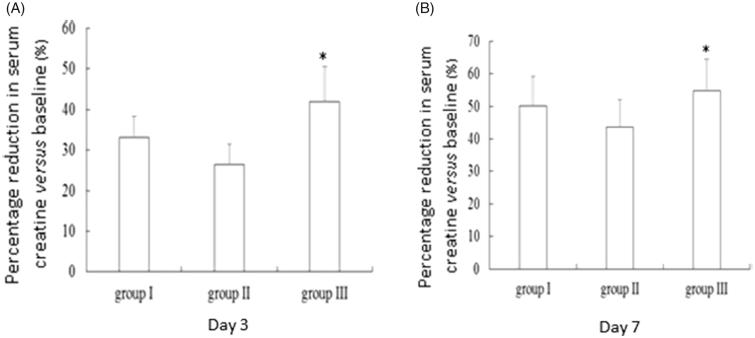
Percentage reduction in serum creatinineversus baseline in patients who developed stage III AKI following wasp stings. Day 3 (A) and day 7 post therapy (B). *p = 0.005.

Serum creatinine at day 3 post-therapy was 452.9 ± 133.1 μmol/L for group I, 595.8 ± 108.2 μmol/L for group II, and 475.6 ± 84.7.1 μmol/L for group III (Table 2). There was a statistically significant difference in serum creatinine among the three groups (*F* = 5.47, *p* = 0.0091). Serum creatinine was significantly higher in group II than group I (*p* = 0.015) and group III (*p* = 0.017). Moreover, group III experienced 41.9% reduction in serum creatinine *versus* baseline, which was significantly higher than that for group I (33.1%) and group II (26.5%) (*p* = 0.005) (Figure 2(A)). Similarly, a statistically significant difference was observed in serum creatinine at day 7 among the three groups (*F* = 9.28, *p* = 0.0007). Serum creatinine was significantly higher in group II (457.6 ± 82.9 μmol/L) than group I (337.6 ± 70.9 μmol/L, *p* ＝ *0*.001) but significantly higher than group III (370.6 ± 49.0 μmol/L, *p* ＝ 0.017). Moreover, group III experienced 54.8% reduction in serum creatinine *versus* baseline, which was significantly higher than that for group I (50.1%) and group II (43.6%) (*p* = 0.001) (Figure 2(B)).

### Secondary outcomes

#### Intra-hospital mortality and cure rate

One (1/13, 7.7%) patient in group I died; four patients were completely recovered and discharged from hospital and eight patients showed improvement and at 6 months of follow-up, were fully recovered, with a cure rate of 92.3% (12/13). Two (2/13, 15.4%) patients in group II died of respiratory failure, four patients fully recovered and discharged from hospital, seven patients improved and were discharged from hospital (Table 3). At 6 months of follow-up, two patients fully recovered while five patients still had mild proteinuria and/or hematuria, with a cure rate of 46.2% (6/13). Two (2/14, 14.3%) patients in group III died, five were fully recovered and seven improved and were discharged from hospital. At 6 months of follow-up, three patients fully recovered and four patients had mild proteinuria and/or hematuria, with a cure rate of 57.1% (8/14). There was no statistical difference in mortality rate among the three groups (*χ*^2^ = 0.4144, *p* = 0.813). On the other hand, statistically significant difference in the cure rate was observed among the three groups (*χ*^2^ = 6.6707, *p* = 0.036).However, there was no statistically significant difference in the cure rate between groups II and III (*p* = 0.427).

**Table 3.** Intra-hospital mortality and clinical outcome.

	Death	Improvement	Recovery
Group I	1	0	12
Group II	2	5	6
Group III	2	4	8

#### Time to polyuria and APACHE II score

**Figure 3.**
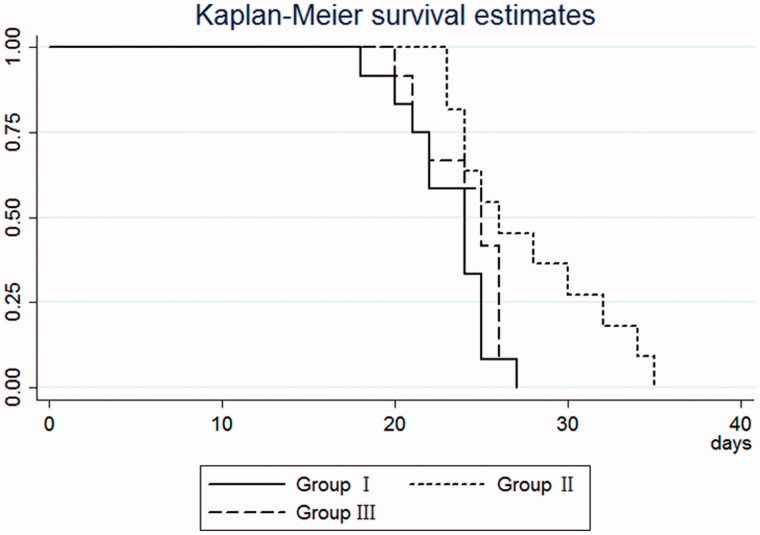
The Kaplan–Meier curve for time to return to polyuriain patients who developed stage III AKI following wasp sting.

The time to polyuria was 23.1 ± 2.5 days for group I, 27.6 ± 4.5 days for group II, and 24.1 ± 2.4 for group III (Table 4). A statistically significant difference was observed in time to polyuria among the three groups (*F* = 6.22, *p* = 0.005). The time to polyuria was significantly longer in group II than group III (*p =* 0.043) and group I (*p* = 0.008), respectively (Figure 3).

**Table 4.** Changes in APACHE II scores and time to polyuria ($\bar{x}\pm s$).

	APACHE II score		Time to polyuria
	Day 3	Day 7	
Group I	16.6 ± 1.6	11.8 ± 1.8	23.1 ± 2.5
Group II	19.4 ± 2.1	14.4 ± 2.3	27.6 ± 4.5
Group III	17.1 ± 2.2	12.1 ± 2.0	24.1 ± 2.4

**Figure 4.**
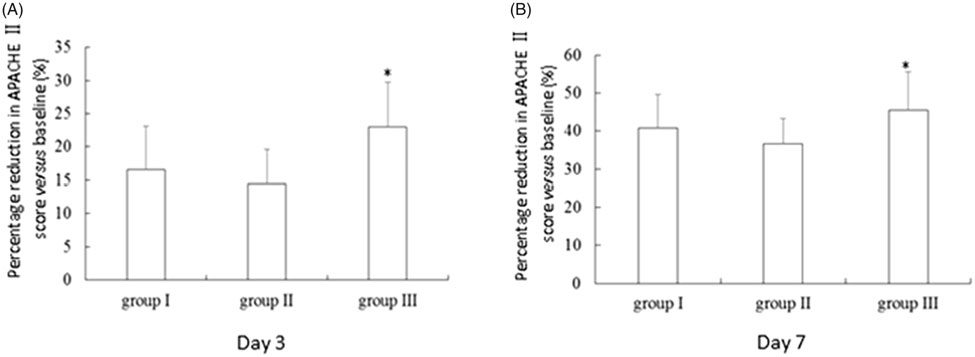
Percentage reduction in APACHE II score versus baseline in patients who developed stage III AKI following wasp stings. Day 3 (A) and day 7 post therapy (B). *p = 0.005.

The APACHE II score at admission was comparable between groups II and III, which was both markedly higher than that of group I (*p* < 0.05) (Table 1).At day 3 post-therapy, the APACHE II score was 16.6 ± 1.6 for group I, 19.4 ± 2.1 for group II, and 17.1 ± 2.2 for group III. A statistically significant difference in APACHE II score was observed among the three groups (*F* = 6.30, *p* = 0.0049). The APACHE II score was significantly higher in group II than group I (*p* = 0.008) and group III (*p* = 0.034). Moreover, group III had the greatest percentage reduction (23.0%) in APACHE II score at day 3 post-therapy *versus* baseline, which was significantly higher than that for group I (16.6%) and group II (14.5%) (*p* = 0.009) (Figure 4(A)). A statistically significant difference was observed in serum creatinine at day 7 among the three groups (*F* ＝ 5.65, *p* ＝ 0.0079). The APACHE II score was significantly higher in group II (14.4 ± 2.3) than group I (11.8 ± 1.8, *p ＝* 0.015) and group III (12.1 ± 2.0, *p ＝* 0.036). Moreover, group III experienced 45.5% reduction in APACHE II score *versus* baseline, which was significantly higher than that for group I (40.7%) and group II (36.6%) (*p* = 0.013) (Figure 4(B)).

#### Safety

Group I had 46 episodes (30.67%) of hypotension and 32 episodes (21.33%) of cardiac arrhythmia (Table 5). Group II had 118 episodes (62.96%) of hypotension and 63 episodes (38.89%) of cardiac arrhythmia and group III had 62 episodes (39.74%) of hypotension and 35 episodes (22.44%) of cardiac arrhythmia. There was a statistically significant difference in the rate of hypotension among the three groups (*χ*^2^ = 35.3282, *p =* −0.0001) and cardiac arrhythmia rate (*χ*^2^ = 15.2934, *p* = 0.0001) among the three groups. Group III had significantly lower rates of hypotension (*χ*^2^ = 17.1555, *p* = 0.0001) and cardiac arrhythmia (*χ*^2^ = 10.0904, *p* = 0.001) than group II.

**Table 5.** Safety outcomes, *n* (%).

	Hypotension	Cardiac arrhythmia
Group I, *n* = 150	46 (30.67)	32 (21.33)
Group II, *n* = 162	118 (62.96)	63 (38.89)
Group III, *n* = 156	62 (39.74)	35 (22.44)

## Discussion

SLEDf has been considered to be a cost effective alternative to continuous renal replacement therapy, but its use in managing wasp stings induced AKI has not been reported. The current study demonstrated that for patients aged 60 years and above, SLEDf was superior to HD in treating stage III AKI associated with wasp stings, with significantly reduced time to normal serum creatinine and experienced 41.9% reduction in serum creatinine *versus* baseline compared to 26.5% for HD.

Wasp stings may cause AKI that mainly manifests pathologically as acute renal tubule necrosis and/or acute interstitial nephritis, leading to renal failure. The current study showed that patients aged 60 years and above had markedly lower mean arterial blood pressure and urine volume and higher baseline serum creatinine and APACHE II scores than those aged less than 60 years, suggesting that elderly wasp sting victims (60 years old) have more severe illness than younger victims (<60 years old). Importantly, SLEDf was superior to HD in treating stage III AKI of elderly wasp stings victims, with significantly more rapid return to normal serum creatinine and greater reduction in serum creatinine *versus* baseline. Wu et al. showed that the presence of renal function impairment in severe AKI patients when discharged from the hospital adversely impacted on the long-term renal outcome [13].However, we observed no statistical difference in either mortality rate or cure rate of elderly patients undergoing HD and those receiving SLEDf, suggesting that faster recovery of serum creatinine by SLEDf did not translate into benefit in mortality rate or cure rate. The lack of benefits in mortality or cure rate, on the other hand, may be due to the small sample size of the study population. However, a previous randomized study found that different modes of HD had no significant impact on the prognosis of severe AKI patients [14].

SLEDf is a novel hybrid renal replacement technique that provides sustained HD (>8 h *versus* 4 h in conventional HD) and continuous blood purification and improves removal mid-sized and large molecules from the plasma by combining dispersive and convective clearance modes. Zsom et al. showed that the duration of a single session of HD therapy was associated with the clearance rate [15]. Because the duration of a single session of SLEDf was longer than that of HD, SLEDf may be more effective in clearing low molecule weight toxins and inflammatory factors.

Compared to conventional HD, SLEDf has a lower blood and dialysate flow rate and clear solutes and water in a sustained manner, slowly reducing the amount of wasp venoms in the blood and allowing time for reaching equilibrium between extracellular and intracellular electrolytes. Longer dialysis session achieves a smaller ultrafiltrate volume per unit time, avoiding hemodynamic instability. We showed that, compared to patients undergoing HD, patients receiving SLEDf had significantly shorter time to polyuria and faster recovery of serum creatinine and lower APACHE II scores 3 days post-treatment, indicating that SLEDf was superior in promoting recovery of renal function in elderly wasp sting victims who developed stage III AKI. Our study is the first report of the efficacy of SLEDf in elderly AKI patients suffering from wasp stings. Consistently, it has been shown that SLEDf significantly improved renal function of sepsis patients who developed AKI [16]. In addition, we found that SLEDf was safer than HD with markedly lower rates of hypotension and cardiac arrhythmia, suggesting that SLEDf exerted lesser hemodynamic impact in wasp sting victims with AKI than HD. Other investigators also demonstrated excellent hemodynamic stability in patients with acute renal failure [17].

The current study has several limitations. Because of its retrospective nature, the study cannot establish a causal relationship. Furthermore, therapeutic interventions were not uniform for all wasp victims, which is more reflective of the real world setting for the healthcare coverage area of the hospital. Meanwhile, we have established work flowchart at hospital to minimize variations of care for wasp victims. In addition, the study did not take into considerations of possible effects of seasonal variations and stings by different wasps. However, because the hospital serves a mountainous area, patients are typically victims of the same type of wasp. Meanwhile, wasp stings mainly occur in the fall season (September–November) of each year in the region and seldom occur outside of the fall season. Therefore, wasp type variation or seasonal variations are unlikely to be confounding factors.

In summary, our study demonstrates that elderly wasp victims have more severe illness than younger wasp victims and SLEDf is safe and superior to HD in recovery of renal function of elderly wasp victims. Our findings, however, await confirmation of prospective randomized study involving a larger population size.
